# Selective Synthesis of Oligosaccharides by Mechanochemical Hydrolysis of Chitin over a Carbon‐Based Catalyst

**DOI:** 10.1002/anie.202214229

**Published:** 2022-11-23

**Authors:** Hirokazu Kobayashi, Yusuke Suzuki, Takuya Sagawa, Makoto Saito, Atsushi Fukuoka

**Affiliations:** ^1^ Komaba Institute for Science Graduate School of Arts and Sciences The University of Tokyo 3-8-1 Komaba Meguro-ku, Tokyo 153-8902 Japan; ^2^ Institute for Catalysis Hokkaido University Kita 21 Nishi 10 Kita-ku, Sapporo Hokkaido 001-0021 Japan; ^3^ Graduate School of Chemical Sciences and Engineering Hokkaido University Kita 13 Nishi 8, Kita-ku Sapporo Hokkaido 060-8628 Japan; ^4^ Department of Industrial Chemistry Faculty of Engineering Tokyo University of Science 6-3-1 Niijuku Katsushika-ku, Tokyo 125-8585 Japan; ^5^ Showa Denko K.K. 1-13-9 Shiba Daimon Minato-ku, Tokyo 105-8518 Japan

**Keywords:** Biomass, Carbon Catalysts, Chitin, Mechanochemistry, Oligosaccharides

## Abstract

Oligosaccharides possess fascinating functions that are applicable in a variety of fields, such as agriculture. However, the selective synthesis of oligosaccharides, especially chitin‐oligosaccharides, has remained a challenge. Chitin‐oligosaccharides activate the plant immune system, enabling crops to withstand pathogens without harmful agrichemicals. Here, we demonstrate the conversion of chitin to chitin‐oligosaccharides using a carbon catalyst with weak acid sites and mechanical milling. The catalyst produces chitin‐oligosaccharides with up to 94 % selectivity in good yields. Monte‐Carlo simulations indicate that our system preferentially hydrolyzes larger chitin molecules over oligomers, thus providing the desired high selectivity. This unique kinetics is in contrast to the fact that typical catalytic systems rapidly hydrolyze oligomers to monomers. Unlike other materials carbons more strongly adsorb large polysaccharides than small oligomers, which is suitable for the selective synthesis of small oligosaccharides.

## Graphical abstract

An activated carbon catalyst having weak acid sites hydrolyzes chitin to chitin‐oligosaccharides selectively in the presence of mechanical milling. Our system preferentially cleaves large molecules over small oligomers, thus maximizing the selectivity and yield of chitin‐oligosaccharides. The product mixture is a promising plant biostimulant for environmentally friendly agriculture.

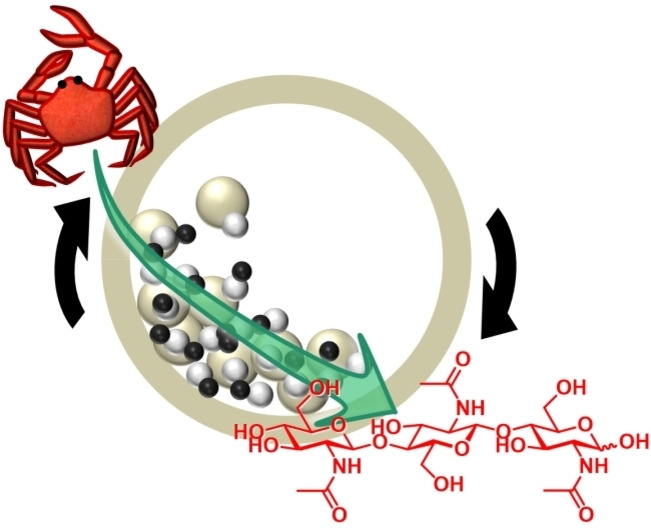

## Introduction

Oligosaccharides are bioactive agents utilized in many organisms. As is well known in blood‐type distinction, cells identify themselves by glycans on the surface, which determines if immune systems attack the tissue.^[1]^ Human milk oligosaccharides work as prebiotics to culture useful intestinal flora and as decoys to block the binding sites of toxic microbes so that infants can maintain good health.^[2]^ Plants use oligosaccharides to modulate their immune systems.^[3]^

**Figure 1:**
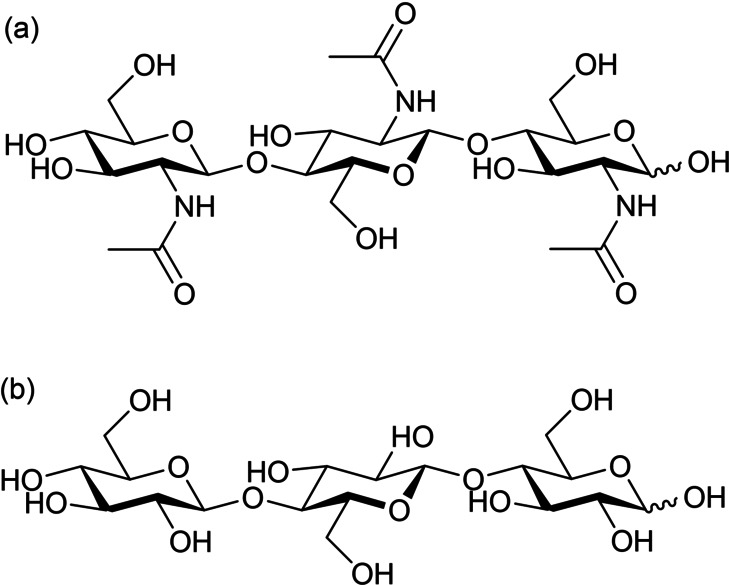
Structures of a) chitin‐ and b) cello‐oligosaccharides. Trimers are depicted as examples.

Oligosaccharides have fascinating functions and may be applied as biostimulants in agriculture.[^4^, ^5^] Some agrochemicals show adverse effects on the environment. For example, neonicotinoid pesticides can impair honey bees’ essential abilities to survive.[^6^, ^7^] To establish environmentally benign agriculture with high productivity, we need alternatives to conventional agrochemicals that protect vegetables and fruits from diseases and pests. A potential methodology is to improve the resistance of plants to microbes. In this regard, chitin‐oligosaccharides and cello‐oligosaccharides (Figure 1), which are β‐1,4‐oligomers of *N*‐acetylglucosamine (NAG) and glucose, respectively, can activate the immune systems of plants.^[3]^ If plants are infected with fungi, they perceive this by detecting particular compounds derived from the pathogens. Such compounds are called pathogen‐associated molecular patterns (PAMPs). The detection of PAMPs promotes the expression of genes that enhance the immune system. Chitin‐oligosaccharides work as PAMPs since they have structures similar to sugar oligomers on the cells of microbes.^[8]^ Therefore, the addition of chitin‐oligosaccharides to plants can stimulate the immune system. Furthermore, if plant bodies are damaged, cello‐oligosaccharides appear as fragments. Plants detect the molecules as damage‐associated molecular patterns (DAMPs), which leads to the expression of immune‐related genes.[^9^, ^10^] Cello‐oligosaccharides with polymerization degrees of 3–6 are especially effective.[^9^, ^10^] The enhanced immune system could eliminate pathogens even without fungicides. Moreover, the oligosaccharides may facilitate the growth of plants.^[5]^ These types of stimulants, the so‐called elicitors, will be essential in future agriculture.

The two oligosaccharides are, however, in short supply. The major issue is that their selective synthesis, especially chitin‐oligosaccharides, is difficult.^[11]^ A possible synthetic route is the hydrolysis of chitin, but enzymatic hydrolysis gives only the dimers as the major products, and thus bioactive oligomers (trimer and larger) are not available.[^12^, ^13^] For thermocatalytic systems, the dilute acid hydrolysis of chitin results in cleavage of both glycosidic and amide bonds in water, and the successive hydrolysis of oligomers to monomers occurs rapidly.[^14^, ^15^] Therefore, the reaction system provides low selectivity for chitin‐oligosaccharides. Although carbon catalysts having weak acid sites convert cellulose to cello‐oligosaccharides in high selectivity in a semi‐batch flow system,^[16]^ the catalysts do not hydrolyze chitin under similar reaction conditions. Another approach is polymerization of monosaccharides, but it produces randomly linked oligomers.[^17^, ^18^]

Recently, mechanochemical methods have emerged as effective tools for green chemical synthesis.^[19]^ They function for the hydrolysis of cellulose and chitin; the synergy of mechanical milling and acid catalysts cleaves glycosidic bonds of sugar polymers to produce oligosaccharides.[^20^, ^21^, ^22^, ^23^, ^24^, ^25^, ^26^, ^27^] Most previous studies used mineral acid catalysts, where the separation of products and catalysts was difficult. In addition, the use of mineral acids produced a significant amount of by‐products that could affect the bioactivity.[^22^, ^23^, ^24^] Meanwhile, solid acid catalysts used in the literature showed much lower activity than mineral acids.[^20^, ^27^] Accordingly, the selective production of oligosaccharides with active solid catalysts will make the products more attractive for practical applications. Herein, we demonstrate the selective synthesis of chitin‐oligosaccharides from chitin over a carbon‐based solid catalyst named AC‐Air. Using a planetary ball‐mill, weak acid sites on the catalyst show much higher activity than previous catalysts toward the hydrolysis of chitin to chitin‐oligosaccharides with minimum by‐product formation.

## Results and Discussion

We observed high activity of AC‐Air in the hydrolysis of chitin in a planetary milling system, in which the selectivity for the chitin‐oligosaccharides reached over 90 %. The results and reaction conditions are summarized in Table 1. In the control experiment with no catalyst for 12 h, 8 wt % of the chitin sample became soluble in water (entry 1). Since the original chitin was insoluble, the solubilization ratio can be regarded as a conversion value of chitin to produce all the soluble compounds. The soluble fraction contained NAG (0.1 % carbon‐based yield, denoted %C), its oligomers (1.2 %C) and by‐products such as humins. Accordingly, the non‐catalytic reaction was ineffective. We prepared a carbon catalyst called AC‐Air, which had 0.90 mmol g^−1^ of carboxylic groups and 0.84 mmol g^−1^ of phenolic groups, by baking a commercial activated carbon in air at 425 °C (details are available in the Supporting Information). AC‐Air increased yields of NAG to 2.8 %C and oligosaccharides to 32 %C (entry 2; HPLC trace: Figure S1). The oligomer yield was 27 times higher than that in the non‐catalytic reaction. Kaolinite, a state‐of‐the‐art material among previously reported solid catalysts, was reported to accelerate the hydrolysis reaction only three times compared to the non‐catalytic reaction.^[27]^ Therefore, the activity of AC‐Air is outstanding. Moreover, the selectivity for oligosaccharides (yield/solubilization ratio=32 %/34 %) was as high as 94 %. The oligomer fraction contained not only dimer (4.7 %C) but also larger oligomers (trimer 5.9 %C, tetramer 6.5 %C, pentamer 5.2 %C, hexamer and larger 9.7 %C). This is an obvious advantage over enzymatic reactions that produce only the dimer. The detailed structures of the oligomers are discussed in a later section.

**Table 1:** Mechanocatalytic hydrolysis of chitin with solid catalysts.

Entry	Substrate	Catalyst	Yield of products^[a]^/%C		Solubilization ratio^[b]^/%
			NAG	Oligosaccharides (selectivity^[c]^/%)	
1	Chitin	–	0.1	1.2 (15)	8.2
2	Chitin	AC‐Air	2.8	32 (94)	34
3^[d]^	Chitin	AC‐Air	8.3	66 (92)	72
4	Chitin	AC	0.1	6.2 (56)	11
5	Chitin	h‐BN	4.9	9.1 (−)^[g]^	–^[g]^
6	Chitin	Kaolinite	1.3	14 (67)	21
7	Chitin	H‐ZSM5	1.6	9.3 (40)	23
8	Chitin	H‐MOR	1.3	9.5 (30)	32
9	Chitin	H‐beta	1.2	10 (42)	24
10	Chitin	SiO_2_‐Al_2_O_3_	0.6	7.1 (26)	27
11^[e]^	Chitin	H_2_SO_4_	7.3	62 (65)	95
12^[d]^	Cellulose	AC‐Air	3.8^[f]^	67 (93)	72

Reaction conditions: Fritsch P‐6, 250 mL alumina pot and 100 g of alumina balls with 5 mm diameter, 500 rpm, 10 min cooling after every 10 min of reaction, total reaction time 12 h, temperature inside ca. 40–50 °C, chitin 5.00 g, catalyst 4.00 g (S/C=1.25). The amount of catalyst was optimized prior to these experiments (Table S1). [a] Yield is on the basis of moles of carbon. [b] Ratio of water‐soluble fraction in the product. [c] Total yield of oligosaccharide / solubilization ratio. [d] Reaction time 48 h. [e] S/C=4.0, 2 h. [f] Glucose. [g] Not available because a significant fraction of the catalyst became soluble after the reaction.

Notably, this reaction produced no acetic acid, indicating no hydrolysis of the amide groups in chitin (−NHCOCH_3_+H_2_O→−NH_3_^+^+CH_3_COO^−^). This feature is in sharp contrast to aqueous‐phase reactions that hydrolyze both glycosidic and amide bonds.^[14]^ Ball collisions in the milling process produce subnano to nano Newton order tensile and compressive forces, which activate the glycosidic bonds for cleavage.[^28^, ^29^, ^30^] Interestingly, the forces do not accelerate the hydrolysis of amide.^[30]^ The rate‐determining step in the acid hydrolysis of amides is the addition of water,^[30]^ which gains little benefit from the milling forces.

The yield of oligosaccharides increased to 66 %C by extending the reaction time to 48 h, while maintaining 92 % selectivity (entry 3). The catalyst was reusable after washing with dilute HCl to remove the adsorbed amines originally contained in chitin (Figure S2). The high catalytic performance of AC‐Air is also observed in the hydrolysis of cellulose; the catalyst afforded 93 % selectivity for oligosaccharides with 67 %C yield (entry 12). These results are clear evidence of the significance of this catalytic system.

The merit of the mechanochemical process is shown by comparison with aqueous‐phase thermal reactions. We have previously reported that a carbon material with weak acid sites hydrolyzes cellulose to glucose and cello‐oligosaccharides in 90 %C yield in water at 180 °C.[^31^, ^32^] Using similar aqueous‐phase reaction conditions, AC‐Air converted chitin to NAG and oligomers in only 1.8 %C yield in total. Chitin is highly recalcitrant,^[33]^ even more than cellulose, and the mechanical forces produced by ball‐milling are essential to achieve the high‐yielding synthesis of chitin‐oligosaccharides. The mechanical forces likely produce tight contact between AC‐Air and chitin. Moreover, the tensile and compressive forces activate the glycosidic bonds of chitin as mentioned above.[^28^, ^30^] The two factors synergistically accelerate the hydrolysis of chitin.

We tested typical solid acids in the hydrolysis of chitin (Table 1). The non‐oxidized activated carbon (AC), which had only 0.08 mmol g^−1^ of carboxylic groups and 0.08 mmol g^−1^ of phenolic groups, was clearly less active (entry 4, oligomer yield 6.2 %C in total) than AC‐Air (32 %C yield). The difference indicates that surface weak acids produced by air oxidation are active. Although strong acids have often been employed for the hydrolysis of polysaccharides, weak acids including carboxylic and phenolic groups can efficiently cleave glycosidic bonds.[^34^, ^35^, ^36^] Hexagonal boron nitride (h‐BN), having lower acidity than AC‐Air,[^37^, ^38^] produced a small amount of products (entry 5, oligomer 9.1 %C). This material became partially soluble in water after the reaction due to exfoliation.^[39]^ In our hands, kaolinite gave a lower yield of products (NAG 1.3 %C, oligosaccharides 14 %C, entry 6) than AC‐Air. Proton‐type zeolites and SiO_2_‐Al_2_O_3_ showed low activity (oligomer <10 %C, entries 7–10). Accordingly, a carbon material with many weak acid sites is uniquely active for this reaction among the solid catalysts tested.

A characteristic of carbon materials is the different driving force for the adsorption of chitin and cellulose molecules. Typical solid acid catalysts are oxides, and their ionic nature favors electrostatic interactions for adsorbing substrates. However, polysaccharides have rigid intra‐ and inter‐molecular hydrogen bonds, thus providing few chances for oxides to interrupt hydrogen bonds for the adsorption. In contrast, carbons can adsorb the polysaccharides by CH‐π interactions.[^40^, ^41^] Specifically, the polycyclic aromatic face of carbon interacts with C−H groups on the axial faces of the polysaccharides by dispersion forces. The adsorption becomes stronger by increasing the molecular size of saccharides.[^41^, ^42^] As a result, the driving force allows for the rapid adsorption of large cellulose molecules even with molecular weights of 22 000.^[42]^ Moreover, carbon materials are more flexible than metal oxides, and this property may enable easy contacts between the catalyst and solid polysaccharides. Weak acid sites on the catalyst surface can then hydrolyze glycosidic bonds of the adsorbed molecules.^[42]^ We propose that these characteristics make AC‐Air more active for the hydrolysis reactions.

**Figure 2:**
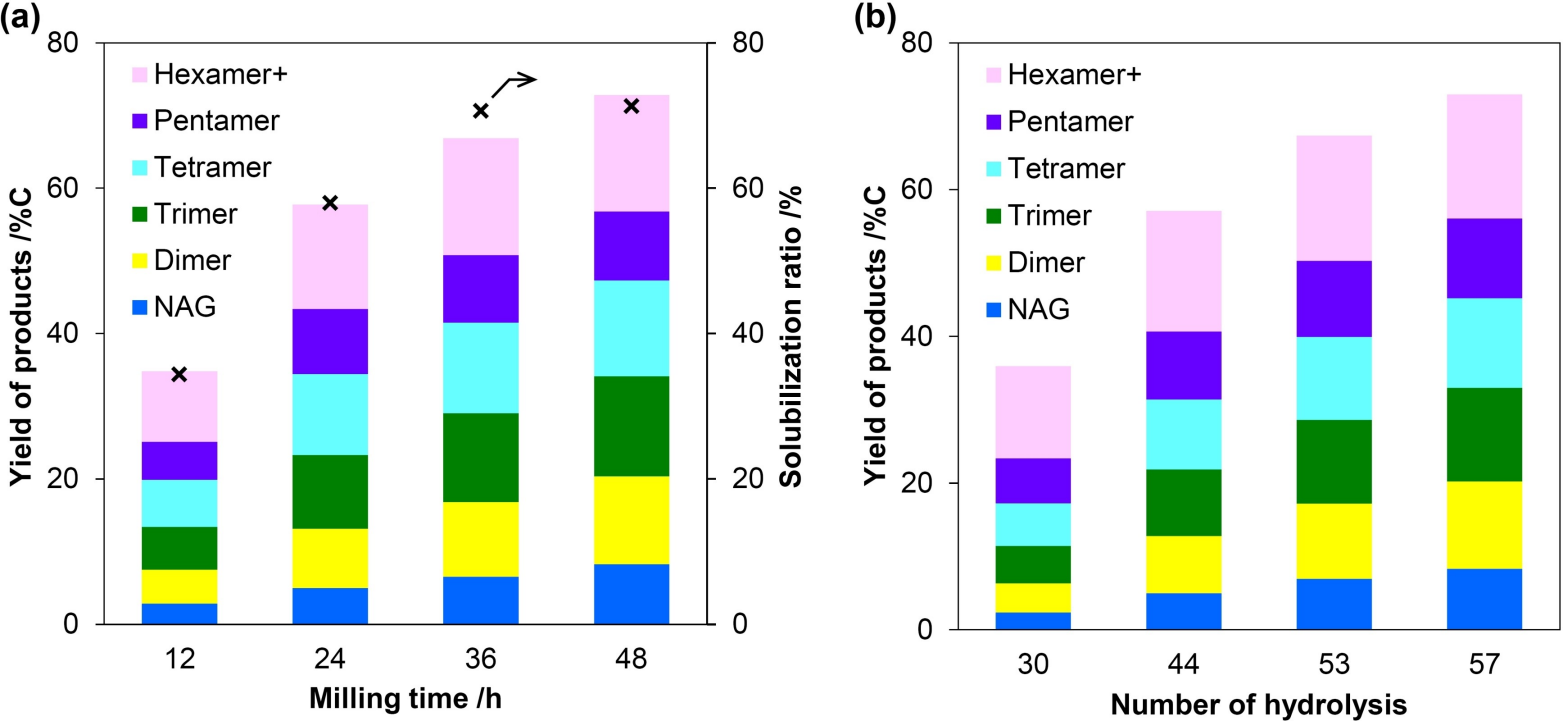
a) Time course of mechanocatalytic hydrolysis of chitin by AC‐Air. S/C=1.25. b) Monte Carlo simulation for random hydrolysis of 200‐mer. Number of hydrolysis was optimized to give a similar total yield of products to the actual data at each reaction time in the time course. The size (200 mer) of chitin was large enough to obtain probabilistic product distributions. In (a), “hexamer+” indicates the total yield of hexamer and larger oligomers. To determine the value for (b), we used the total yield of hexamer and heptamer, since larger oligomers have very low solubility in water.

While AC‐Air gives good yields of oligosaccharides, it is unclear whether it preferentially hydrolyzes long‐ or short‐chain saccharides. This is a decisive factor in maximizing the yield of oligosaccharides. For example, if a catalyst more rapidly hydrolyzes small oligosaccharides than large polysaccharides, the reaction gives a low yield of oligomers. To clarify this issue, we analyzed the time course of the mechanocatalytic hydrolysis of chitin over AC‐Air (Figure 2a). The solubilization ratio for chitin and the yield of oligosaccharides both increased with reaction time and gradually leveled off. The ratio of products was nearly constant within 48 h, indicating limited successive hydrolysis of oligosaccharides to NAG. To understand this behavior, we applied Monte Carlo simulations that virtually hydrolyzed a chitin molecule. In detail, starting from a 200‐mer chitin as a model of our substrate, the simulation randomly cleaved glycosidic bonds at designated times, where the random number was produced by the Mersenne Twister mt19937.^[43]^ Repeating the simulation 100 000 times provided a probabilistic product distribution (convergence behavior Figure S3). Random scission (Figure 2b) afforded a product distribution similar to the actual results (Figure 2a), whereas constraints on the choice of cleaving points in the simulation increased deviations from the experimental results (Figure S4). These results show that AC‐Air can cleave glycosidic bonds of chitin molecules without influence of the molecular size. This means that the hydrolysis rate of a chitin molecule (*v*) increases linearly with increasing polymer size: *v* ∝ (number of glycosidic bonds in a chitin molecule)=(polymerization degree−1). In the mechanochemical system, any size of chitin molecules in the solid particle can be present at the substrate‐catalyst interface. The molecules are adsorbed on the catalyst and are subjected to uniform mechanical forces by ball collisions during milling. Therefore, the hydrolysis probability is proportional to the number of glycosidic bonds in the molecule. This characteristic is in contrast to aqueous‐phase reactions using mineral acids or Amberlyst that rapidly hydrolyze small oligomers.[^44^, ^45^] In the aqueous phase, soluble small oligomers more easily gain access to the catalysts than insoluble large polymers, and undergo rapid hydrolysis. Consequently, the mechanocatalytic system can reduce the successive hydrolysis of small oligomers, thus giving a high yield and selectivity of oligosaccharides.

**Figure 3:**
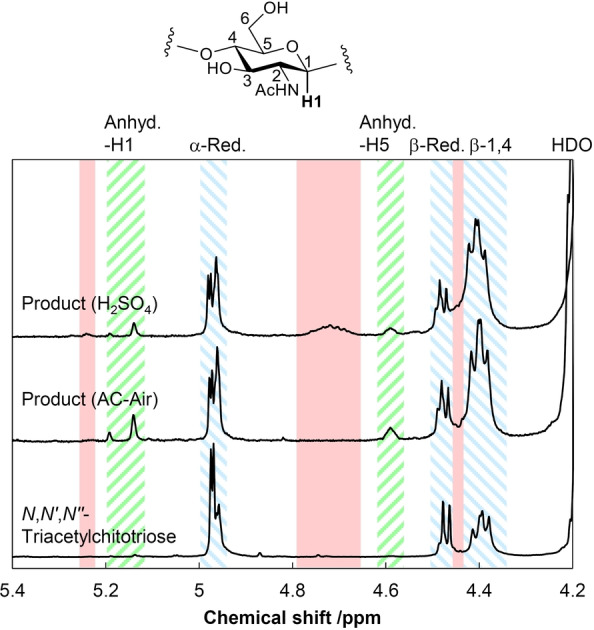
^1^H NMR spectra of hydrolysates and standard trimer in D_2_O‐DMSO‐d6 (1 : 3) mixture. Blue diagonal line: H1 for linear chitin‐oligosaccharides including a small amount of NAG (α‐reducing end, β‐reducing end, β‐1,4‐glycosidic bond); green diagonal line: H1 and H5 for anhydride forms of reducing ends in any oligosaccharide; red fill: H1 for other oligosaccharides with unidentified structures. The chemical shifts for anhydrides were determined using 1,6‐anhydro‐β‐*N*‐acetylglucosamine.

We determined detailed structures of the oligosaccharides produced in the mechanocatalytic hydrolysis of chitin under the optimized reaction conditions (the products in entry 3 of Table 1). Previously, D_2_O solvent was employed for the ^1^H NMR analysis of chitin‐oligosaccharides,^[24]^ but in this work we found that the solvent peak (4.79 ppm) overlapped with those for the by‐products. Therefore, shifting the HDO peak to a higher field by adding deuterated dimethylsulfoxide (DMSO‐d6) enabled us to precisely analyze the products. Among the proton peaks, it is convenient to focus on the hydrogen atoms bonding to C1 (Figure 3), named H1, because they appear separately at lower magnetic fields. We detected peaks for chitin‐oligosaccharides at 4.97 ppm (α reducing end), 4.47 ppm (β reducing end) and 4.39 ppm (inner unit and non‐reducing end). Three additional peaks at 5.1–5.2 and 4.58 ppm were derived from the anhydride form of the reducing ends (Figure S5), but their content was only 5 %. We also confirmed the presence of chitin‐oligosaccharides and anhydrides by LC/MS analysis (Figure S6). Accordingly, the H1 peak area assignable to NAG and proper chitin‐oligosaccharides accounts for 95 % of the total H1 peak area.

For comparison, we performed a mechanocatalytic hydrolysis of chitin with H_2_SO_4_ under the optimized conditions for this liquid catalyst (Table 1, entry 11), since this mineral acid is the most frequently used in the conversion of biomass.[^21^, ^22^, ^23^, ^24^] This reaction gave a 95 % solubilization ratio and 62 %C yield of oligomers. The selectivity for oligomers was only 65 %, much lower than that for AC‐Air (92 %; entry 3). The by‐product formation was verified by NMR analysis, giving additional broad peaks at ca. 4.7 ppm as highlighted in red (Figure 3). DEPT and 2D NMR techniques indicated that the new peaks are assignable to H1 in by‐products with uncertain structures (Figures S7–S9). It is known that the reaction with H_2_SO_4_ causes branching reactions between two polysaccharide molecules.^[22]^ This comparison demonstrates that AC‐Air is more selective than the typical catalysts for the production of chitin‐oligosaccharides.

The reaction of chitin with H_2_SO_4_ takes place on the surface of chitin or in the bulk phase, which is true with any liquid acid catalyst. In contrast, the carbon‐based catalyst hydrolyzes chitin at the interface between the carbon and chitin. This alteration of the reaction environment is a characteristic specific to solid catalysts. The different reaction environment changes physicochemical properties related to chemical reactions such as the dielectric constant and interactions with surrounding molecules. Intermolecular side reactions with two chitin molecules are possibly decreased on the carbon surface, compared to the liquid acid system. Thus, we speculate that not only the weak acidity of the carbon but also the different environment around the reaction centers improve the selectivity.

## Conclusion

AC‐Air hydrolyzes chitin to chitin‐oligosaccharides selectively in the presence of mechanical milling. Active sites of the catalyst are weak acid sites introduced by air oxidation. The catalyst is significantly more active than previous solid acid catalysts and provides higher selectivity than sulfuric acid for producing chitin‐oligosaccharides. Our Monte‐Carlo simulations indicate that AC‐Air can cleave glycosidic bonds of chitin molecules at a similar rate without relation to molecular size. This kinetics leads to the selective production of oligosaccharides. Different from other materials carbons easily adsorb large polysaccharides, and therefore AC‐Air can hydrolyze large polymers to small oligomers more selectively and quickly than other solid catalysts. We hope the oligosaccharide mixtures produced by this method will be utilized in the agriculture sector. It is notable that AC‐Air does not hydrolyze chitin in the aqueous phase but functions under mechanochemical conditions. This insight may be informative in developing a conversion method for recalcitrant solid polymers.

## Conflict of interest

The authors declare no conflict of interest.

1

## Supporting information

As a service to our authors and readers, this journal provides supporting information supplied by the authors. Such materials are peer reviewed and may be re‐organized for online delivery, but are not copy‐edited or typeset. Technical support issues arising from supporting information (other than missing files) should be addressed to the authors.

Supporting Information

## Data Availability

The data that support the findings of this study are available from the corresponding authors upon reasonable request.
