# Supplementary material for: Selective Synthesis of Oligosaccharides by Mechanochemical Hydrolysis of Chitin over a Carbon‐Based Catalyst
Source: Angew Chem Int Ed Engl. 2022 Nov 23;62(3):e202214229. doi: 10.1002/anie.202214229 (PMC10099807; doi:10.1002/anie.202214229)
Supplement: Supplementary file 1 — Supporting Information [file ANIE-62-0-s001.pdf]

Angewandte Chemie
Eine Zeitschrift der Gesellschaft Deutscher Chemiker

Supporting Information

Selective Synthesis of Oligosaccharides by Mechanochemical Hydrolysis of Chitin over a Carbon-Based Catalyst

H. Kobayashi, Y. Suzuki, T. Sagawa, M. Saito, A. Fukuoka**

Materials and methods

Catalyst preparation

The air-oxidized activated carbon, named AC-Air, was prepared from commercial steam-activated carbon (herein called AC, Ajinomoto Fine Techno, grade BA). AC (4.00 g) was spread thinly on a Pyrex dish (ø130) placed in an electric furnace. The temperature was elevated to 120 °C at 5 °C min^{-1} and kept at that temperature for 2 h to remove moisture. The sample was again heated at a rate of 5 °C min^{-1} to 425 °C and kept under that condition for 10 h. After cooling, 2.0 g of AC-Air was obtained.

Acidic oxygenated functional groups on AC-Air were determined by the Boehm titration method[S1] using 50 mM NaOH, Na_2CO_3 and $NaHCO_3$ aqueous solutions. After ion exchange with the bases under an inert gas atmosphere for 24 h, the mixture was filtered through filter paper of quantitative grade (Advantec, 5A). Hydrochloric acid (50 mM) was added to a portion of the filtrate to make it acidic and CO_2 was removed under a reduced pressure of 500 hPa with sonication for 5 min. Afterwards, a colorimetric titration was performed with 50 mM NaOH in the presence of methyl orange as an indicator, where the endpoint was pH 4.0. The use of methyl orange minimized the influence of CO_2 (apparent pK_a 6.4) contamination[S2] on the titration.

Reaction and product analysis

A 5.00 g quantity of chitin (Wako; moisture content 6.7 wt%) or cellulose (Aldrich, Avicel PH-101; moisture content 2.6 wt%) was milled with a catalyst (4.00 g) in the presence of alumina balls (ø5, 100 g) in an alumina pot (250 mL) at 500 rpm by a planetary mill (Fritsch, P-6). Physisorbed water was used for the hydrolysis reactions. The milling procedure included a 10-minute interval after every 10 minutes of milling to maintain the pot temperature below 333 K. The catalysts used were AC, AC-Air, hexagonal boron nitride (h-BN; Maruka, AP-20S), kaolinite (Aldrich, kaolin, K7375), SiO_2/Al_2O_3 (JGC, Si/Al = 3.3), H-ZSM-5 (Tosoh, HSZ-822HOA; Si/Al = 12), H-MOR (Tosoh, HSZ-660HOA; Si/Al = 15), H-beta (Tosoh,

HSZ-931HOA; Si/Al = 14) and SiO_2 (Catalysis Society of Japan, JRC-SIO-9A).

After the milling process, a portion of the recovered products (100 mg as biomass) was dispersed in water by sonication for 10 min, and separated into liquid and solid residue by filtration using a hydrophilic polytetrafluoroethylene membrane (Advantec; pore size 0.1 μm). The solution was analyzed with a high-performance liquid chromatograph (Shimadzu, Prominence) equipped with a refractive index detector (RID). Three SB802.5HQ columns (Shodex; ø8, 300 mm length) were connected in series for good separation of oligosaccharides. The column temperature was 55 °C and the mobile phase was pure water. The amounts of products were calculated by an absolute calibration method. Product yield in this work was based on the number of carbon atoms (%C). The solid residue was weighed after drying at 110 °C to calculate the solubilization ratio of the product.

Products were identified with a nuclear magnetic resonance (NMR) spectrometer (JEOL, ECX-600; ^{1}H 600 MHz) in the presence of 2,2-dimethyl-2-silapentane-5-sulfonic acid sodium salt (DSS) as an internal standard.

Anhydrides of *N*-acetylglucosamine (Fig. S1) were synthesized as references according to the literature.[S3] NAG (0.50 g) was added in sulfolane (25 mL), and the solution was heated at 160 °C for 2 h. After cooling the mixture, the products were isolated by HPLC equipped with a Rezex RPM-monosaccharide Pb++ column (70 °C, eluent H_2O).

1,6-Anhydro-β-*N*-acetylglucosaminopyranoside (AHAGP): ^{1}H NMR (600 MHz, D_2O): δ 2.00 (s, 3H, CH_3), 3.62 (s, 1H, H3), 3.71 (s, 1H, H4), 3.74–3.76 (m, 2H, H2 and H6), 4.15 (d, J = 7.6 Hz, 1H, H6), 4.62 (d, J = 4.8 Hz, 1H, H5), 5.40 (s, 1H, H1). ^{13}C NMR (151 MHz, D_2O): δ 24, 55, 68, 73, 74, 79, 103, 176.

1,6-Anhydro-β-*N*-acetylglucosaminofuranoside (AHAGF): ^{1}H NMR (600 MHz, D_2O): δ 2.00 (s, 3H, CH_3), 3.82 (m, 2H, H5 and H6), 4.33 (d, J = 13.7 Hz, 1H, H6), 4.38–4.41 (m, 3H, H2, H3 and H4), 5.07 (s, 1H, H1). ^{13}C NMR (151 MHz, D_2O): δ 24, 63, 66, 69, 78, 82, 106, 177.

Computations

Monte Carlo simulations for the hydrolysis of polysaccharides were performed with a self-made program written in C++. The source code was compiled with gcc version 6.3.0 included in MinGW. The simulation randomly cleaved glycosidic bonds for a designated number of times in a polymer molecule with a polymerization degree of 200. The random numbers were generated with the Mersenne Twister, mt19937.[S4] The program contained an option that does not degrade oligosaccharides of a designated size or smaller. We changed the number of iterations for the hydrolysis simulation from 1 to 1,000,000 to observe the convergence behavior, and found that more than 10,000 times was suitable (Fig. S3). Therefore, the number was set at 100,000 times as a sufficiently high value.

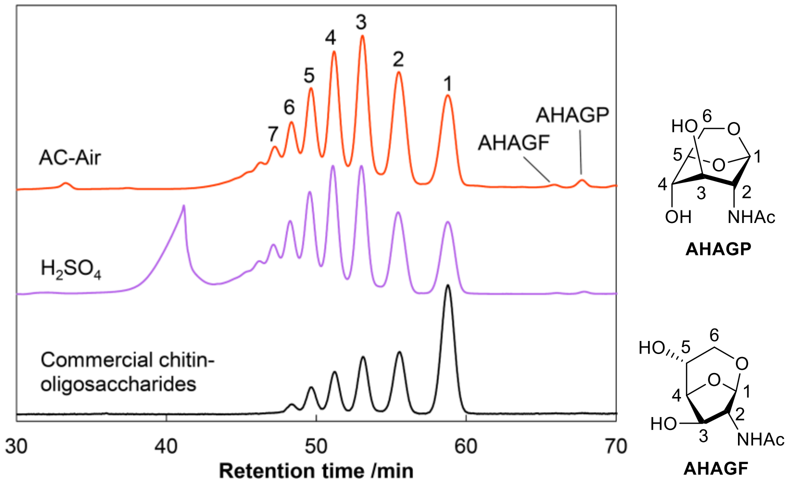

Figure S1. HPLC traces for hydrolysis of chitin and standard.

The numbers indicate the peaks for NAG to heptamer.

AHAGP: 1,6-anhydro-β-*N*-acetylglucosaminopyranoside.

AHAGF: 1,6-anhydro-β-*N*-acetylglucosaminofuranoside.

The chromatograms were shifted up to 0.4 min to align the retention time for NAG at the same position in the three traces. The column condition slightly affected the retention time. Note: when the formation of by-products such as anhydrides and branched ones was significant, the peak positions for oligomers shifted due to similar retention times for some of the by-products. Additionally, the peaks for oligomers produced by H_2SO_4 were slightly shifted.

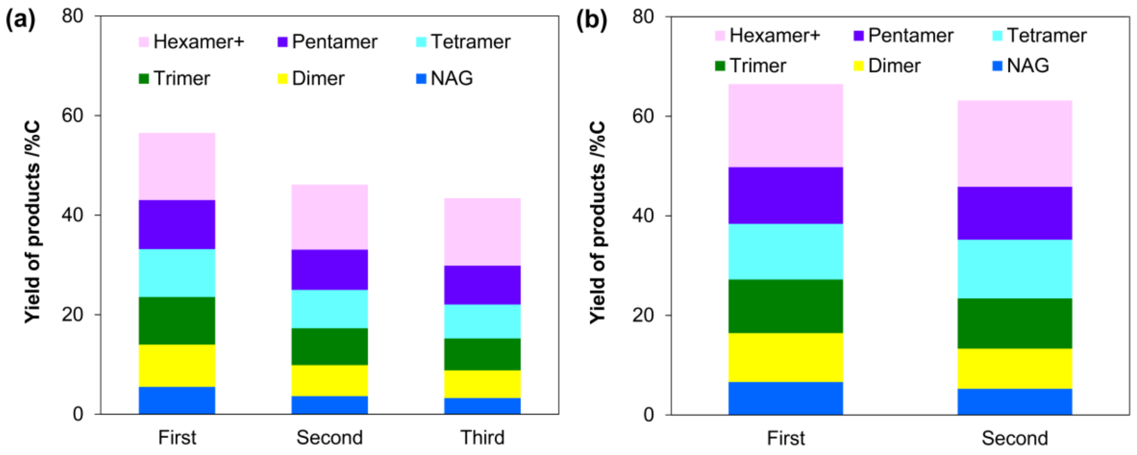

Figure S2. Reuse of AC-Air in hydrolysis of chitin. S/C = 1.25, reaction time 24 h. (a) Catalyst was reused as is. (b) Catalyst was reused after washing with HCl.

The durability of AC-Air was evaluated in the mechanocatalytic conversion of chitin for 24 h (Fig. S2a). After a reaction, the entire sample was dispersed in water to collect soluble products. Fresh chitin substrate was added to the solid residue to compensate for the amount of soluble products before continuing the next cycle of the mechanocatalytic treatment. In other words, the same amount of chitin was used in the repeated reactions. The first run gave a 51% yield of oligosaccharides, which was similar to that observed in the time-course study (Fig. 2, 24 h, 53%), *viz.*, good reproducibility. The catalytic activity was slightly decreased, with a yield of 42% in the second run and 40% in the third. This was attributed to the neutralization of acidic groups of the catalyst with free amino groups contained in the original chitin. Therefore, we reused AC-Air after washing with 0.1 M HCl (Fig. S2b). Indeed, adsorbed species were removed from the catalyst surface, and the catalytic activity was nearly maintained.

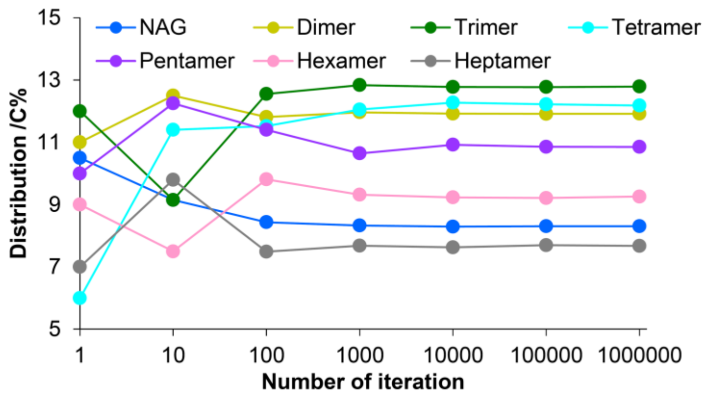

Figure S3. Effect of number of iterations in Monte Carlo simulations.

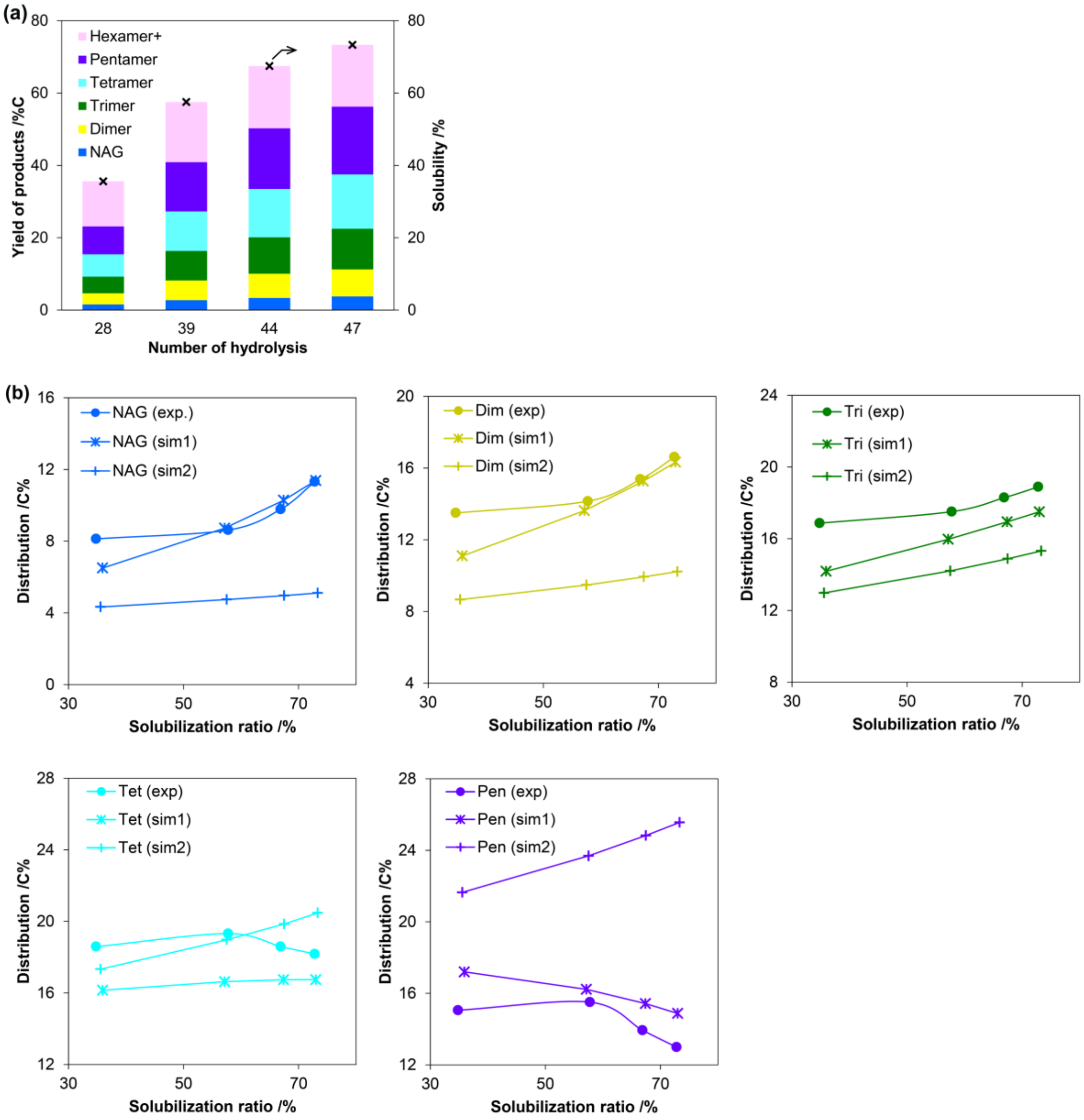

Figure S4. (a) Monte Carlo simulations for random hydrolysis of 200-mer with restriction that pentamer and smaller products were not hydrolyzed. (b) Product distributions for actual experiments (exp), simulation with no restriction (sim1) and (a) (sim2). “NAG” to “Pen” indicates monomer to pentamer. Distribution = carbon-based yield of the product / solubilization ratio.

The sim1 nicely followed the actual experiments. In contrast, the sim2 gave much lower selectivity for NAG but higher for pentamer. The selectivity for pentamer continuously increased in sim2, but the actual experiment showed an opposite trend.

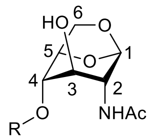

Figure S5. Equivalent structure to AHAGP can be formed in oligomers at reducing end.

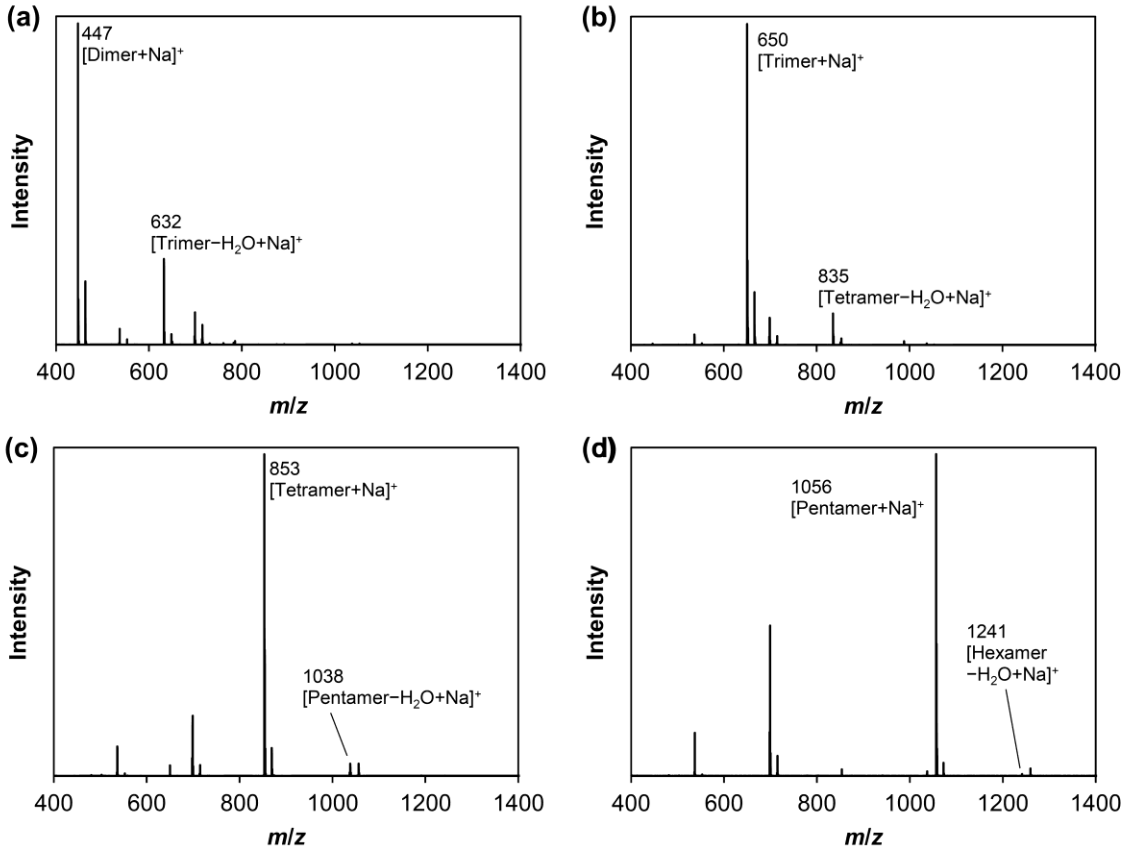

Figure S6. MALDI/TOF-MS analysis of HPLC fraction for chitin hydrolysate. Retention times for chitin-oligosaccharides for (a) dimer, (b) trimer, (c) tetramer and (d) pentamer. Intensity was normalized.

The spectra show that small quantities of anhydrides exist in the fraction of one-unit smaller chitin-oligosaccharides. For (a), the peak for the anhydride of the trimer is relatively large due to low detection sensitivity of the dimer. The MALDI system ionizes larger molecules more efficiently.

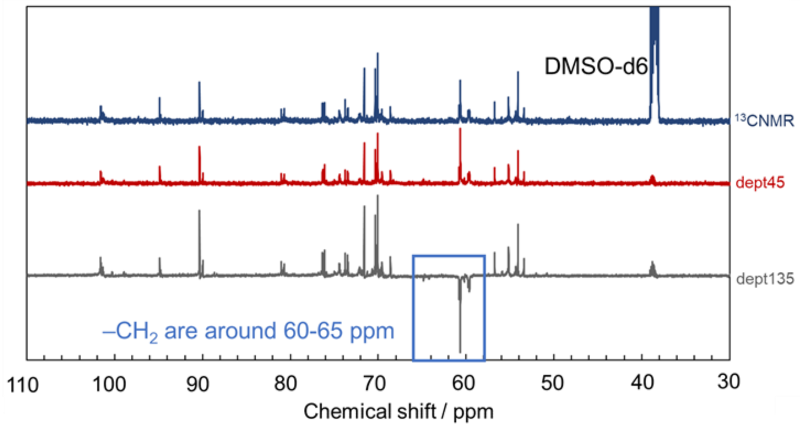

Figure S7. DEPT spectra for hydrolysate of chitin hydrolysis with H_2SO_4 by ball-milling.

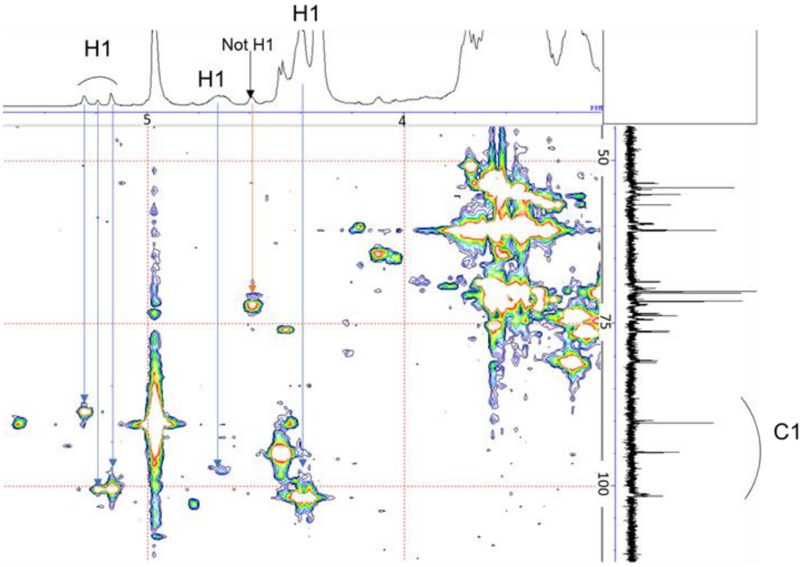

Figure S8. HMQC spectrum for hydrolysate of chitin hydrolysis with H_2SO_4 by ball-milling. The position of HDO is slightly different from that in Fig. 3 due to a slight difference in the concentration of DMSO.

Peaks at 4.7 and 5.1–5.2 ppm show a correlation with C1, and thus they are assigned to H1.

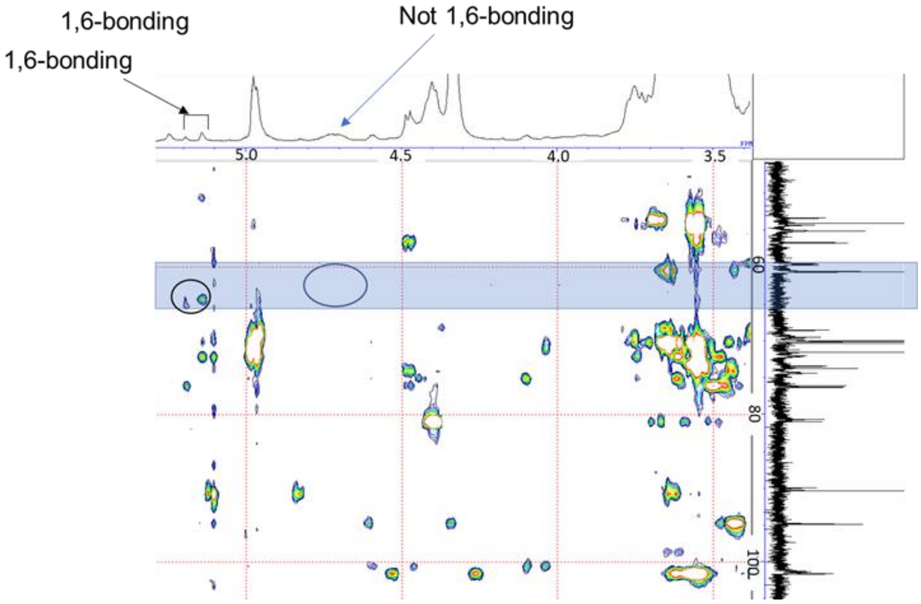

Figure S9. HMBC spectrum for hydrolysate of chitin hydrolysis with H_2SO_4 by ball-milling. The position of HDO is slightly different from that in Fig. 3 due to a slight difference in the concentration of DMSO.

The blue zone shows the range of C6 peaks. The peaks at 5.1–5.2 ppm show correlations with C6, indicating the presence of 1,6-bonds. In contrast, the broad peak at 4.7 ppm does not show any correlation, and therefore the structure is ambiguous.

Table S1. Effect of S/C on hydrolysis of chitin by AC-Air.

S/C	Yield of products /%C					Solubilization ratio /%
	NAG	Dimer	Trimer	Tetramer+[b]	Oligomers in total	
No catalyst	0.1	0.1	0.2	0.8	1.2	8.2
6.48	0.2	0.3	0.5	1.9	2.8	0.4
2.50	0.8	1.7	2.2	11	15	15
1.25	2.8	4.7	5.8	21	32	34
0.63	8.6	7.3	5.8	11	24	34

[a]Chitin 5.00 g, 500 rpm, 12 h. [b]Tetramer and larger oligomers.

A previously reported method named mix-milling involved ball-milling similar carbon catalysts and polysaccharides together, but it only created good physical contact without hydrolysis.[S5] The result does not conflict with the mechanocatalytic hydrolysis in this work. First, the treatment time is much shorter (<2 h)[S6] than those for the mechanocatalytic reactions. More importantly, when we used the same amount of AC-Air as required in the mix-milling (S/C = 6.48), the mechanocatalytic hydrolysis for 12 h produced only a 2.8 %C yield of oligomers. The mechanocatalytic reaction needs more catalyst to drive the solid–solid reaction. An increase in the catalyst amount from S/C = 6.48 to 1.25 drastically improved the yield of oligomers (2.8 %C to 32 %C). In the presence of only a small amount of catalyst, we speculate that the catalyst surface is easily fully covered with substrate and partially hydrolyzed products, which likely suppresses further hydrolysis.

References

[S1] H. P. Boehm, *Carbon* **1994**, *32*, 759-769.

[S2] S. L. Goertzen, K. D. Thériault, A. M. Oickle, A. C. Tarasuk, H. A. Andreas, *Carbon* **2010**, *48*, 1252-1261.

[S3] H. Kaga, M. Enomoto, H. Shimizu, I. Nagashima, K. Matsuda, S. Kawaguchi, A. Narumi, *Molecules* **2020**, *25*, 1944.

[S4] M. Matsumoto, T. Nishimura, *ACM Trans. Model. Comput. Simul.* **1998**, *8*, 3-30.

[S5] H. Kobayashi, M. Yabushita, T. Komanoya, K. Hara, I. Fujita, A. Fukuoka, *ACS Catal.* **2013**, *3*, 581-587.

[S6] H. Kobayashi, H. Kaiki, A. Shrotri, K. Techikawara, A. Fukuoka, *Chem. Sci.* **2016**, *7*, 692-696.
